# Exercise intervention in middle-aged and elderly individuals with insomnia improves sleep and restores connectivity in the motor network

**DOI:** 10.1038/s41398-024-02875-2

**Published:** 2024-03-22

**Authors:** Rongrong Chen, Shilei Wang, Qinzi Hu, Ning Kang, Haijiang Xie, Meng Liu, Hongyu Shan, Yujie Long, Yizhe Hao, Bolin Qin, Hao Su, Yongchang Zhuang, Li Li, Weiju Li, Wei Sun, Dong Wu, Wentian Cao, Xiaoqin Mai, Gong Chen, Dongmin Wang, Qihong Zou

**Affiliations:** 1 Department of Psychology, Renmin University of China, Beijing, China; 2 Center for MRI Research, Academy for Advanced Interdisciplinary Studies, Peking University, Beijing, China; 3 Beijing City Key Lab for Medical Physics and Engineering, Institution of Heavy Ion Physics, School of Physics, Peking University, Beijing, China; 4 Center for Magnetic Resonance Imaging Research & Key Laboratory of Applied Brain and Cognitive Sciences, School of Business and Management, Shanghai International Studies University, Shanghai, China; 5 Institute of Population Research, Peking University, Beijing, China; 6 Department of Physical Education, Peking University, Beijing, China; 7 Sports Coaching College, Beijing Sports University, Beijing, China; 8 The School of Sports Science, Beijing Sport University, Beijing, China; 9 China Wushu School, Beijing Sport University, Beijing, China; 10 Peking University Hospital, Beijing, China; 11 National Clinical Research Center for Mental Disorders (Peking University Sixth Hospital), Beijing, China

**Keywords:** Neuroscience, Diseases

## Abstract

Exercise is a potential treatment to improve sleep quality in middle-aged and elderly individuals. Understanding exercise-induced changes in functional plasticity of brain circuits that underlie improvements in sleep among middle-aged and older adults can inform treatment of sleep problems. The aim of the study is to identify the effects of a 12-week exercise program on sleep quality and brain functional connectivity in middle-aged and older adults with insomnia. The trial was registered with Chinese Clinical Trial Register (ChiCTR2000033652). We recruited 84 healthy sleepers and 85 individuals with insomnia. Participants with insomnia were assigned to receive either a 12-week exercise intervention or were placed in a 12-week waitlist control condition. Thirty-seven middle-aged and older adults in the exercise group and 30 in the waitlist group completed both baseline and week 12 assessments. We found that middle-aged and older adults with insomnia showed significantly worse sleep quality than healthy sleepers. At the brain circuit level, insomnia patients showed decreased connectivity in the widespread motor network. After exercise intervention, self-reported sleep was increased in the exercise group (*P* < 0.001) compared to that in the waitlist group. We also found increased functional connectivity of the motor network with the cerebellum in the exercise group (*P* < 0.001). Moreover, we observed significant correlations between improvement in subjective sleep indices and connectivity changes within the motor network. We highlight exercise-induced improvement in sleep quality and functional plasticity of the aging brain.

## Introduction

Insomnia is the most common sleep disorder and the second most common mental disorder [1], which is characterized by subjective difficulties in initiating and maintaining sleep, frequent or prolonged awakenings, or early-morning awakening with an inability to return to sleep, along with substantial distress and impairments in daytime functioning [2, 3]. Insomnia is highly prevalent in older adults [4, 5] and imposes severe economic burdens on individuals and society [6]. The main treatment for insomnia includes psychological/behavioral therapy and pharmacological treatment or a combination of the two [6, 7]. The limited efficacy, side effects, and high costs of these therapies make the development of alternative approaches essential.

Exercise intervention has been recognized as a potential treatment to improve sleep [8–10]. Accumulating evidence has revealed that medium- to long-term exercise programs can improve sleep quality [11], sleep efficiency [12], and sleep duration [12–14] as well as reduce self-reported sleep latency [13, 15] and sleep disturbance [12]. The most frequent exercise intervention includes aerobic and resistance exercise. Tai Chi, a type of mind-body aerobic exercise, has recently been widely researched as a method of alleviating sleep problems in the older population [12, 16–18]. Tai Chi emphasizes a gentle, balanced, and stable rhythm. It has been recommended as a suitable exercise for middle-aged and older adults. In a recent study, Siu and colleagues reported that Tai Chi improved insomnia in a large sample of older adults [19]. Resistance training, also called strength training or weighted training, involves the progressive use of loads, movements, or velocities to improve muscle strength and endurance [20, 21]. The effect of resistance training on sleep has also been demonstrated in older adults [22]. Combining resistance training with Tai Chi may further promote physical function and sleep compared with Tai Chi exercise or resistance training only [23].

The neural basis of exercise interventions has been extensively mapped in animal models. These studies suggest that physical activity or exercise can lead to morphological and functional changes in the brains of older animals [24]. Exercise interventions promote changes in the motor cortex, cerebellum, striatum, and hippocampus in rats [25]. In humans, aerobic exercise increases functional connectivity (FC) and gray and white matter volumes mainly located in the motor, prefrontal and temporal cortices [26–29]. Although previous studies have suggested the potential of exercise to influence brain plasticity [30, 31], the underlying functional changes in brain areas that explain exercise-induced improvement in insomnia symptoms in middle-aged and older adults remain unclear. Resting-state FC possesses the potential to serve as crucial biomarkers or therapeutic targets for improving insomnia [32, 33]. This method allows the examination of temporal correlations among spatially distinct brain regions, providing insights into the inherent organization and functioning of the brain [32, 34, 35]. Significantly, It has contributed to uncovering the underlying mechanisms for accurately evaluating exercise intervention effectiveness, particularly among the middle-aged and elderly [36].

In the current study, we first identified the neurobiological mechanisms of insomnia via functional magnetic resonance imaging (fMRI) by comparing middle-aged and older individuals with and without insomnia. Given that the motor cortex has been demonstrated to show deficiency in insomnia [37–39], be involved in sleep-wake control [40], and could be modified by exercise intervention [41–43], we focused on the FC of motor network. Specifically, whole-brain FC analysis with the primary motor cortex (M1) as the seed were performed using resting-state fMRI data from 84 healthy sleepers and 85 insomnia volunteers. The middle-aged and older adults with insomnia symptoms were randomly assigned to receive either exercise intervention (EX) or a waitlist control condition (WL). The waitlist condition was adopted to control for the passage of time and assessment in the population of interest [44]. We applied a 12-week exercise intervention program that combined Tai Chi and resistance training to investigate its effects on subjective and objective sleep quality as well as brain FC in middle-aged and elderly individuals with insomnia symptoms. Previous studies have shown a link between poor sleep quality and problems with emotional regulation [45, 46]. Therefore, we also evaluated anxiety and depression before and after the intervention. Thirty-seven middle-aged and older adults in the EX group and 30 in the WL group finished baseline and after-assessments. We predicted that exercise would significantly improve self-reported and objective sleep quality compared to the control condition. We also expected brain FC to recover after exercise. Additionally, we hypothesized that changes in FC would track improvements in insomnia symptoms.

## Methods

### Participants and study design

Participants were recruited through advertisements in the neighborhood of Peking University campus. The inclusion criteria were as follows: (1) Chinese, (2) right-handed individuals, (3) 45-80 years old, and (4) Mini-Mental State Exam (MMSE) score > 25. Individuals with insomnia had to meet at least one of the diagnostic criteria for insomnia disorder in the Diagnostic and Statistical Manual of Mental Disorders, fifth edition (DSM-5) [47]. Healthy controls (HCs) were enrolled if they had self-reported satisfactory sleep [48]. Exclusion criteria were as follows: (1) history of psychiatric or neurological illness; (2) history of psychoactive substance abuse; (3) presence of serious heart problems, liver disease, kidney disease, infectious disease, endocrine problems or cancer; (4) employment as a shift worker or a recent trans-meridian trip (in the last three months); (5) contraindications to MRI; or (6) use of drugs that affect sleep within two weeks before the study.

Baseline data were acquired from HCs after their enrollment. Participants with insomnia symptoms were screened via face-to-face structured interviews, and those who met the criteria were invited for baseline data collection. The insomnia patients were sequentially allocated to the 12-week EX group or WL group according to the order of enrollment, with the first half of the participants in the EX group and the other in the WL group. After the 12-week intervention, participants were invited for sleep evaluation and MRI assessment, with the same data acquisition parameters as at baseline (see Supplementary material for details).

This study adhered to the Transparent Reporting of Evaluations with Nonrandomized Designs (TREND) guidelines [49]. All procedures contributing to this work comply with the ethical standards of the relevant national and institutional committees on human experimentation and with the Helsinki Declaration of 1975, as revised in 2008. All procedures involving human subjects/patients were approved by the Committee for Protecting Human and Animal Subjects School of Psychological and Cognitive Sciences (Peking University, China; approval number 2019-03-07). All participants provided written informed consent. The intervention was registered on the Chictr.org.cn website before the start of enrollment (ChiCTR2000033652, http://www.chictr.org.cn/showproj.aspx?proj=54773). There was no change in the protocol during the study period.

### Exercise intervention

We adopted an exercise intervention designed by our team, which has a multidisciplinary background, including clinical doctors, sports researchers, and Tai Chi instructors. This protocol consists of a warm-up and formal training. The traditional Tai Chi elements are performed throughout the process. The warm-up exercise was Tai Chi Chan Si Gong. The training session included Zhuang Gong (including Tai Chi Zhuang and Tai Chi Ball), Chen-style 8-form Tai Chi, and resistance training. The details of the intervention are provided in the Supplementary material (Supplementary text and Table S1). The exercise intervention was performed in community-based groups under the guidance of experienced and qualified instructors. All the EX participants engaged in three sessions a week, each lasting one hour. The WL participants maintained normal activities without intervention during the 12 weeks. After finishing all assessments, the WL group received a video about the 12-week exercise intervention protocol to improve insomnia through self-practice.

### Baseline evaluations

During the recruitment process, demographic information was collected, including age, sex, and years of education. Baseline information was also collected on the following variables: MMSE score, body mass index (BMI), history of drug use/abuse and history of mental illness or neurodegenerative diseases. In addition, all participants were asked to complete the International Physical Activity Questionnaire (IPAQ) to measure their weekly exercise.

### Outcomes measures

Subjective sleep quality was measured with the Insomnia Severity Index (ISI) [50] and Pittsburgh Sleep Quality Index (PSQI) [51]. Participants were also asked to complete the Self-Rating Anxiety Scale (SAS) and Self-Rating Depression Scale (SDS) as indices for negative emotion, which is closely related to insomnia symptoms [52]. We used a single electroencephalography-based wearable forehead sleep recorder (UMindSleep, EEGSmart Co., Ltd.) to monitor objective sleep [53]. Sleep recording will be performed twice for each participant, with the first night used for adaptation and the data from the second night used for analysis. If the signal quality on the second night is poor (e.g., the device is accidentally disconnected from the forehead), a third night of recording will be performed. Continuous automatic identification of the 4 sleep stages (wakefulness, light sleep, deep sleep, and rapid eye movement sleep) will be performed from the captured EEG recordings using built-in algorithms every 30 seconds during sleep and visually verified by an experienced technician. The duration of each sleep stage, total sleep duration, percentage in each sleep stage, and wakefulness after sleep onset (WASO) will be extracted as objective sleep indices capturing sleep quality.

MRI data were acquired using a 3T Siemens Prisma scanner (Siemens Healthineers, Erlangen, Germany) with a 64-channel head coil at the Center for MRI Research, Peking University. Resting-state blood oxygenation level-dependent (BOLD) fMRI data were collected using a gradient echo planar imaging sequence (repetition time (TR) = 2,000 ms, echo time (TE) = 30 ms, flip angle (FA) = 90°, number of slices = 33, slice thickness = 3.5 mm, gap = 0.7 mm, matrix = 64 × 64, and in-plane resolution = 3.5 × 3.5 mm^2^). During data acquisition, participants were asked to lie quietly in the scanner with their eyes open. An MR-compatible camera was used to ensure that participants did not fall asleep. The scanning procedure lasted for 480 s (240 TRs) for each participant. For registration purposes, high-resolution T1-weighted structural images were acquired by using a 3-dimensional magnetization-prepared rapid acquisition gradient-echo sequence (TR = 2530 ms, TE = 2.98 ms, inversion time (TI) = 1100 ms, FA = 7°, number of slices = 192, and voxel size = 0.5 × 0.5 × 1 mm^3^).

The fMRI data were preprocessed using Analysis of Functional NeuroImages (AFNI) [54] and MATLAB (MathWorks). Specifically, the preprocessing steps included the removal of the first five volumes, slice-timing and head-motion correction, spatial normalization (voxel size, 2 × 2 × 2 mm^3^) to the International Consortium for Brain Mapping standard template (ICBM152) using T1 image segment information, nuisance covariate removal, linear trend removal and bandpass filtering (0.009–0.08 Hz). The nuisance covariates included Friston’s 24 head motion parameters [55], mean signals of white matter, cerebral spinal fluid and the whole brain and their first derivatives. Spatial smoothing with a 6-mm Gaussian kernel was then performed.

The bilateral M1 were chosen separately as seeds based on the automated anatomical labeling atlas [56]. We calculated the Pearson’s correlation coefficient between each seed time series and every other voxel in the brain and transformed the correlation coefficients into z values using Fisher’s r-to-z transformation.

The primary outcome following the intervention was the improvement in sleep quality, assessed by subjective sleep questionnaires and objective sleep architecture. The secondary outcome was changes in the FC of the motor network.

### Statistical analyses

A comparison of subjective and objective sleep parameters between individuals with insomnia and HCs was conducted using independent-sample *t* tests in IBM SPSS Statistics 26.0 (IBM Corporation, Armonk, New York). The effect of exercise on sleep parameters was determined with a generalized estimated equation (GEE) model including the factors of group (EX, WL) and time (baseline and week 12) as main effects and baseline as a covariate in SPSS. Bonferroni adjustments were used for multiple comparisons.

Differences in seed-based FC of the left and right M1 between the insomnia patients and HCs were determined using 3dttest++ in AFNI. The effects of age, sex, years of education, weekly exercise, and head motion parameters were controlled in the independent-sample *t* tests. The head motion parameters included the maximum head motion, the number of volumes with framewise displacement larger than 1, and average framewise displacement during the fMRI scan. We used an autocorrelation function (ACF) combined with a voxel­wise threshold of *P* < 0.001 to correct for multiple comparisons [57, 58]. The ACF was implemented in the 3dClustSim tool to determine the cluster size threshold to use for a given voxel­wise threshold, which was estimated using 3dFWHMx based on the residuals from 3dttest++. Correspondingly, a corrected significance level of *P* < 0.05 for the resulting statistical maps was obtained using clusters, with minimum numbers of 229 voxels and 241 voxels for left and right M1 connectivity, respectively, at an uncorrected individual voxel height threshold of *P* < 0.001.

Based on the regions that showed significant differences in left M1 connectivity and right M1 connectivity, we further tested 1) the potential effect of exercise on brain functional indices using GEE (including group and time) in SPSS and 2) the relationship between changes in brain FC (week 12 - baseline) and improvements in sleep parameters in the EX group using Pearson’s correlation analysis in MATLAB.

## Results

### Adherence to the exercise intervention and baseline characteristics of participants

In total, 163 middle-aged and older adults with insomnia symptoms were recruited from 20 March 2021 to 13 December 2021, of which 92 participants met the inclusion criteria and were invited to participate in the intervention, 51 in the EX group and 41 in the WL group. Eighty-five out of the 92 insomnia patients were assessed for the primary and secondary outcomes at baseline (following enrollment), and 67 of them were assessed after 12 weeks (Supplementary Fig. S1). Participants were lost to follow-up for the following reasons: (i) missed one exercise session per week for any reason or (ii) inability to complete the week 12 assessment. In addition, 206 HCs were recruited from 2 October 2021 to 29 October 2022; 122 HCs were excluded for different reasons, and 84 HCs completed the assessments (Supplementary Fig. S1).

The baseline demographic and clinical characteristics of the study participants are presented in the Supplementary material. No significant differences in age, sex, years of education, MMSE score, BMI, or IPAQ score were observed between participants with insomnia group and HCs (Supplementary Table S2), or between the EX and WL groups (Supplementary Table S3). Notably, the EX group showed a higher SDS score than the WL group (*P* < 0.001). In addition, no significant differences in demographic information (age, sex, and years of education) were found between insomnia individuals completed baseline and week 12 assessments (*N* = 67) and those dropped out (*N* = 18) during the intervention, while these adhered participants have a trend of longer rapid eye movement sleep onset latency than those dropped out (*P* = 0.05). No adverse events were observed.

### Differences in sleep parameters and functional connectivity between insomnia and healthy controls

The insomnia patients had significantly worse sleep quality than HCs, with larger ISI and PSQI scores (Insomnia vs. HCs: ISI difference = 14.17; 95% CI, 13.23–15.12; *P* < 0.001 and PSQI difference = 8.23; 95% CI, 7.41–9.04; *P* < 0.001). In addition, the insomnia patients were more affected by negative emotion (SAS and SDS) than the HCs (Insomnia vs. HCs: SAS difference = 11.35; 95% CI, 8.91–13.78; *P* < 0.001 and SDS difference = 14.61; 95% CI, 11.41–17.82; *P* < 0.001). We also found a longer period of WASO and rapid eye movement sleep onset latency (RSOL) in the insomnia group compared to HCs according to the objective sleep measures (Insomnia vs. HCs: WASO difference = 25.53 minutes; 95% CI, 12.79–38.26; *P* < 0.001 and RSOL difference = 23.09 minutes; 95% CI, 0.21–45.96; *P* = 0.048). See Table S2 in the Supplementary material.

**Fig. 1:**
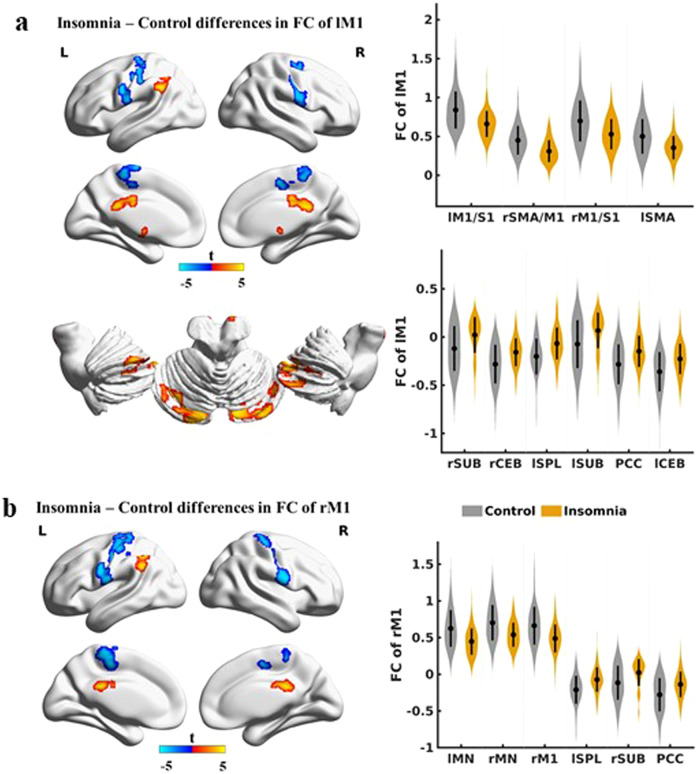
Differences in seed-based FC between insomnia patients and healthy controls. **a** Seed-based FC of the left M1. **b** Seed-based FC of the right M1. *P* < 0.05 corrected. FC functional connectivity, M1 primary motor cortex, S1 primary sensory cortex, SMA supplementary motor area, SUB subcortical regions including caudate putamen and thalamus, CEB cerebellum, SPL superior parietal lobule, PCC posterior cingulate cortex, MN primary motor network (including the bilateral M1), R right, L left.

Using the primary motor cortices as seeds, we observed decreased positive connectivity within the motor network in middle-aged and older adults with insomnia. In addition, the negative connectivity between the M1 and the superior parietal lobule, subcortical areas, posterior cingulate cortex, and cerebellum (CEB) was decreased in participants with insomnia. The findings are consistent with the seeds located in the left (Fig. 1a) and right M1 (Fig. 1b). See Tables S4 and S5 in the Supplementary material.

### Primary outcomes: subjective and objective sleep quality

**Fig. 2:**
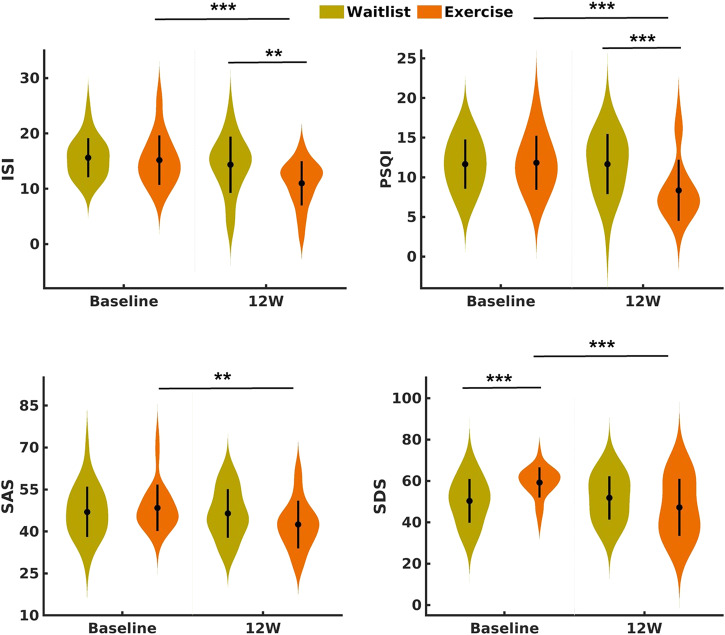
Sleep-related subjective assessments at 2 time points (baseline and week 12) in the waitlist and exercise conditions. ISI Insomnia Severity Index, PSQI Pittsburgh Sleep Quality Index, SAS Self-Rating Anxiety score, SDS Self-Rating Depression score, 12 W week 12.

There was a significant effect of the interaction between group and time on sleep quality, as indexed by decreases in ISI and PSQI scores of the EX group (Baseline vs. week 12: ISI change = 4.19; 95% CI, 2.57–5.81; *P* < 0.001 and PSQI change = 3.49; 95% CI, 2.46–4.51; *P* < 0.001) and nonsignificant changes in those of the WL group. Importantly, the EX group had better sleep quality than the WL group after 12 weeks (EX vs. WL: ISI difference = −3.36; 95% CI, −5.56 to −1.16; *P* = 0.0027 and PSQI difference = −3.32; 95% CI, −5.13 to −1.50; *P* < 0.001) (Fig. 2). We conducted a further analysis of the PSQI scores based on the seven subdimensions (sleep quality, sleep latency, sleep duration, habitual sleep efficiency, sleep disturbances, use of sleep medication, and daytime dysfunction). Our findings revealed significant effects of the interaction between group and time on five out of the seven subdimensions, including sleep quality scores, sleep latency scores, sleep duration scores, habitual sleep efficiency, and daytime dysfunction scores (Supplementary Table S7). Consequently, the exercise intervention demonstrates a comprehensive influence on various sleep symptoms.

The exercise intervention significantly alleviated negative emotion, as reflected by decreased SAS and SDS scores in the EX group at week 12 (Baseline vs. week 12: SAS change = 5.97; 95% CI, 2.69–9.26; *P* < 0.001 and SDS change = 12.05; 95% CI, 7.05–17.06; *P* < 0.001). Using objective sleep monitoring devices, exploratory analysis revealed shorter WASO after 12 weeks of exercise, although this interaction effect was not significant (Fig. 2 and Supplementary Table S7).

### Secondary outcomes: motor network functional connectivity

**Fig. 3:**
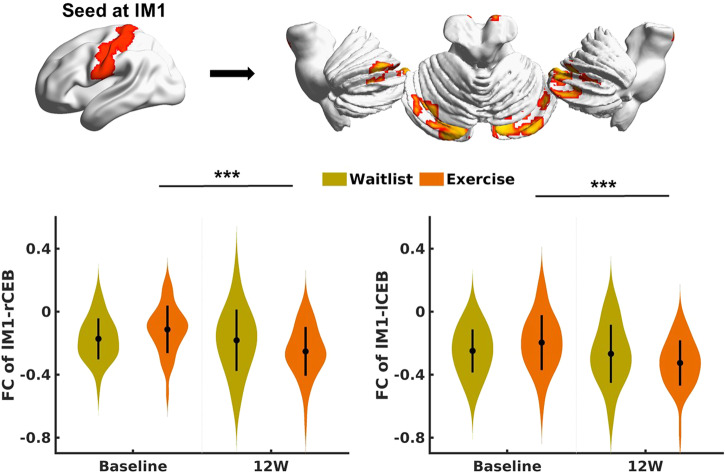
Changes in the FC between the left M1 and bilateral cerebellum after exercise compared with the waitlist condition. FC functional connectivity, M1 primary motor cortex, CEB cerebellum, R right, L left, 12 W week 12.

Significant effects of exercise on the FC between the left M1 and the bilateral CEB were observed (Fig. 3). Post hoc analyses showed that exercise significantly restored the negative connectivity between the left M1 and the bilateral CEB at week 12 (Baseline vs. week 12: lM1-rCEB FC change = 0.14; 95% CI, 0.09– 0.19; *P* < 0.001 and lM1-lCEB FC change = 0.13; 95% CI, 0.07–0.19; *P* < 0.001), which was decreased in insomnia patients compared to WLs (Fig. 3). In contrast, the WL group showed nonsignificant changes.

### Brain-behavior relationship

**Fig. 4:**
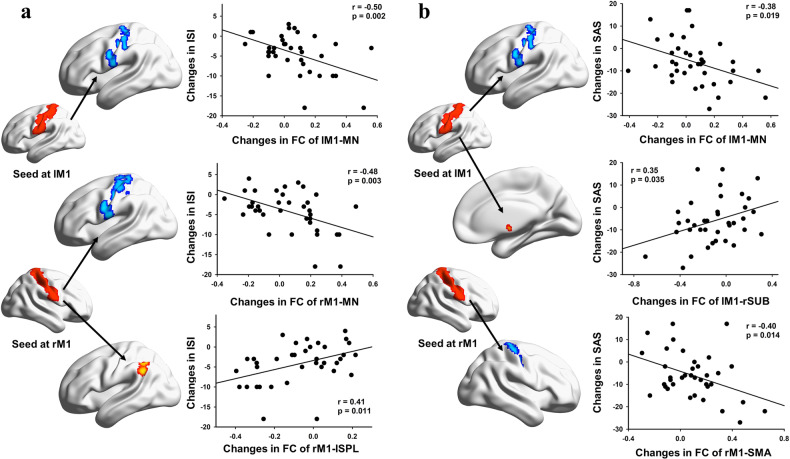
Relationship between changes in self-reported sleep quality and negative emotion and FC in exercise condition. **a** Correlation between ISI scores and seed-based FC. **b** Correlation between SAS scores and seed-based FC. ISI Insomnia Severity Index, SAS Self-Rating Anxiety Scale, FC functional connectivity, M1 primary motor cortex, MN primary motor network (including the bilateral M1), SPL superior parietal lobule, SUB subcortical regions including caudate, putamen, and thalamus, SMA supplementary motor area, R right, L left.

Further exploration of the correspondence between improvements in sleep quality and brain functional plasticity induced by the 12-week exercise program revealed significant correlations between improvements in sleep (indexed by lower ISI scores) and connectivity changes (Supplementary Table S8) within the primary motor network (*r* = −0.50; *P* = 0.002 and *r* = −0.48; *P* = 0.003; Fig. 4a) and between the right M1 and the superior parietal lobule (*r* = 0.41; *P* = 0.011; Fig. 4a). The greater the restoration of motor network connectivity was, the larger the improvement in sleep quality. In addition, alleviation of negative emotion, as shown by decreased SAS scores, was associated with changes in connectivity (Supplementary Table S9) within the primary motor network (*r* = −0.38; *P* = 0.019 and *r* = −0.40; *P* = 0.014; Fig. 4b) and between the right M1 and subcortical regions (*r* = 0.35; *P* = 0.035; Fig. 4b).

### Sensitivity analysis

In light of the observed differences in depression scores between the EX group and the WL group within our sample, we included SDS scores as covariates for a more refined sensitivity analysis. The results substantiated our prior findings, confirming significant effects stemming from the interaction between group and time. We identified a notable impact on sleep quality, as evidenced by reductions in ISI and PSQI scores (*P* = 0.008 and *P* < 0.001), on negative emotion, as indicated by lower SAS scores (*P* = 0.009), and on the connectivity of M1-CEB within the EX group (*P* = 0.003 and *P* = 0.011).

Notably, significant correlations persisted even when accounting for SDS scores as covariates. Specifically, correlations were identified between improvements in sleep (indicated by lower ISI scores) and connectivity changes within the primary motor network (*r* = −0.53; *P* < 0.001 and *r* = −0.46; *P* = 0.005), as well as between the right M1 and the superior parietal lobule (*r* = 0.44; *P* = 0.007). The reduction in negative emotion, as reflected by decreased SAS scores, exhibited associations with changes in connectivity within the M1 (*r* = −0.40; *P* = 0.016 and *r* = −0.39; *P* = 0.017), as well as between the right M1 and subcortical regions (*r* = −0.36; *P* = 0.033).

## Discussion

We found that middle-aged and older adults with insomnia had decreases in both subjective and objective sleep quality. HCs had better self-reported sleep quality (lower ISI and PSQI scores), less negative emotion (lower SAS and SDS scores), and better objective sleep quality (shorter RSOL and WASO values). At the brain circuit level, elderly individuals with insomnia symptoms showed decreased positive FC within the motor network and negative FC of the M1 with the superior parietal lobule, subcortical areas, posterior cingulate cortex, and CEB. A 12-week exercise intervention was performed, and we found that exercise improved insomnia (measured by self-reported sleep quality) and FC. Our results showed that our exercise protocol (combining Tai Chi and resistance training) is an effective and feasible approach to alleviating insomnia symptoms and restoring connectivity of the motor network in middle-aged and older adults.

We identified differences between elderly participants with and without insomnia in terms of subjective and objective sleep quality and negative emotion. Additionally, the WASO and RSOL values, measured with a portable sleep recorder, was different in middle-aged and older adults with insomnia. WASO and RSOL are important objective indicators of sleep quality [59]. Additionally, our findings provided evidence of a neurobiological difference between insomnia patients and HCs. Previous resting-state fMRI studies have found altered FC of the frontoparietal network in insomnia patients aged 21–65 years [60, 61]. People with insomnia aged 22–70 years also had lower variation in FC between the anterior salience network and the left executive-control network [62]. We revealed FC differences in the motor network; FC might be the neurobiological mechanism underlying insomnia among elderly individuals, consistent with the motor theory of sleep-wake control derived from animal models [40] and neuroanatomical changes in the motor system of insomnia patients [39].

Previous studies on exercise interventions for insomnia have indicated that exercise is an effective approach to improve sleep quality. Our findings are consistent with the results of these previous studies [13, 19]. In addition, our study expands upon previous findings in several ways.

First, we used both subjective and objective measures to evaluate insomnia. Many studies have measured only subjective sleep quality (typically with a self-reported sleep questionnaire) before and after the exercise intervention. The objective method in the current study involved electroencephalography recordings of the frontal lobe to accurately infer sleep architecture. The portable sleep recording device allowed participants to finish obtaining recordings at home, which is easier for participants compared to recordings in laboratory conditions. A shortened WASO was observed after exercise training (although there was not a significant interaction between group and time). A previous study observed improvements in subjective WASO after yoga [63] or walking exercise [64]. Our findings, demonstrating improvements in objective WASO, are consistent with these results. Although longer RSOL was observed in insomnia patients than in HCs, the exercise intervention did not affect RSOL. Longer-term exercise interventions and/or other treatments should be explored in future studies.

Second, this is the first study to investigate the effects of a 12-week exercise intervention on FC and insomnia symptoms among elderly individuals. Our data provide preliminary evidence of FC recovery after exercise training in older adults with insomnia. Previous studies have adopted Tai Chi and Ba Duan Jin practice to improve memory, reporting increased FC between the bilateral hippocampus and the medial prefrontal cortex in older adults [65]. Our study using a combined exercise intervention further enhances understanding of FC changes in the motor network after exercise among insomnia individuals. In addition, studies have applied repetitive transcranial magnetic stimulation targeting the M1 for motor rehabilitation and found evidence of improvement in insomnia symptoms after treatment [66, 67].

The overarching model that explains insomnia is the hyperarousal hypothesis [65, 66] which proposes that the pathophysiology of insomnia is associated with the dysregulation of arousal mechanisms [68]. This dysregulation is due to decreased levels of inhibitory neurotransmitters (i.e., γ-aminobutyric acid, adenosine, and serotonin) [69]. In contrast, studies have also indicated increases in levels of inhibitory neurotransmitters in insomnia [68]. In the current study, we found hypoconnectivity in the motor network, which is contrary to the hyperarousal hypothesis. This difference might be due to the heterogeneity of insomnia and comorbid conditions. We carefully identified and excluded middle-aged and older adults with potential comorbid physical and mental conditions in the present study. Another possible explanation is that insomnia in aging population might result from the disruption of the alternating rhythms of wake-promoting and sleep-promoting molecules [70, 71]. Evidence has revealed that several sleep-regulation molecules (i.e., noradrenaline) affect the sleep/wake cycle. Insomnia patients might suffer from lower levels of sleep-promoting molecules than HCs. Exercise might regulate or increase levels of these molecules [72] and influence brain activation. In our study, the hypoactivity in the M1 of insomnia individuals is in accordance with altered levels of inhibitory neurotransmitters. It should be noted that alteration between the M1 and the cerebellum, subcortical regions and the higher-order cortical regions in insomnia were not significantly restored after exercise intervention, although the extent of connectivity changes were associated with the improvement in sleep quality and negative emotion alleviation.

Third, the alterations in FC of M1 and the cerebellum prompt an exploration of its role in the amelioration of insomnia, offering insights into the broader neurological mechanisms influenced by exercise intervention. The increased FC in M1 suggests enhanced communication within the motor cortex, which may be linked to improved motor function and skill acquisition resulting from exercise. Furthermore, the observed enhancement in insomnia symptoms within the study cohort may be attributed to improved sleep-related motor control facilitated by the strengthened connectivity between M1 and cerebellum [73]. The coordination of motor activity during sleep is acknowledged as pivotal in preventing disruptions that may lead to wakefulness [74, 75]. Hence, the optimization of sleep-related motor control through heightened M1-cerebellar connectivity emerges as a compelling explanation for the observed positive impact on insomnia. Another intriguing possibility stems from directed connectivity from the motor cortex to cerebellum, suggesting a neocortical origin of slow waves and its impact on spindles [76]. This implies that the connectivity changes observed may play a role in modulating sleep oscillations, contributing to a more synchronized and efficient sleep pattern.

Fourth, our intervention program combined Tai Chi and resistance training to maximize interventional effects. Tai Chi involves aerobic exercise of light to moderate intensity [77]. Studies have demonstrated moderate-intensity aerobic exercise [78] or resistance training has positive effects on objective and subjective sleep quality and emotion regulation in insomnia individuals [79]. Gupta and colleagues compared the effects of aerobic and resistance training on sleep quality in older adults and revealed that both resistance training and aerobic exercise improved sleep quality [80]. A combination of the two might be more efficacious than engaging in one type of exercise alone.

Limitations of the present study should be noted. First, most of the enrolled participants were female; thus, the generalizability of findings may be limited by the sex ratio. Females are more likely to experience insomnia [81]. In future studies, a balanced sex ratio should be established. Second, we included only a waitlist control group rather than a sham condition, which makes it difficult to interpret the effects of exercise in the EX group, i.e., whether the effect observed is a sham effect or a real intervention effect. Nonetheless, the waitlist control condition could serve as a benchmark against which intervention outcomes of insomnia can be compared. The lack of sham treatment for the control group is clinically meaningful as this indicates the relative advantage (and cost-effectiveness) of including a specific intervention. Third, our findings demonstrate that the exercise intervention significantly alleviated the insomnia symptoms of participants after 12 weeks. However, we did not include further follow-up assessments (for example, at 6 months or 12 months). Future studies are needed to evaluate the long-term effects of exercise interventions. Fourth, there is a noticeable baseline difference in depression scores between the EX group and WL group, with the intervention group exhibiting significantly higher levels of depression. However, even after accounting for depression levels by using SDS score as a covariate in our further analysis, we observed improvements in sleep outcomes. This discrepancy in baseline depression levels introduces a potential confounding factor that may influence the interpretation of the intervention’s impact on insomnia. Future research should consider more balanced baseline characteristics to further explore the effect of exercise intervention on insomnia symptoms.

In conclusion, the present study showed motor network hypoconnectivity in middle-aged and older adults with insomnia. We further demonstrated that an exercise intervention improved insomnia symptoms and motor network connectivity. We highlight the ability to modulate sleep quality and plasticity of the aging brain via exercise intervention.

### Supplementary information

Supplementary materials

## Data Availability

Data are available when required with reasonable request by contacting zouqihong@pku.edu.cn.
