# Supplementary material for: Exercise intervention in middle-aged and elderly individuals with insomnia improves sleep and restores connectivity in the motor network
Source: Transl Psychiatry. 2024 Mar 22;14:159. doi: 10.1038/s41398-024-02875-2 (PMC10959941; doi:10.1038/s41398-024-02875-2)

**Exercise Intervention in Older Adults with Insomnia Improves Sleep and Restores Connectivity in the Motor Network**

Rongrong Chen^1^, MA; Shilei Wang^2,3,4^, MA; Qinzi Hu^2,3^, BA; Ning Kang^5^, BA; Haijiang Xie^6^, BA; Meng Liu^7^, BA; Hongyu Shan^2,3^, MA; Yujie Long^2,3^, MA; Yizhe Hao^2,3^, BA; Bolin Qin, BA^2,3^; Hao Su^8^, PhD; Yongchang Zhuang^9^, MA; Li Li^10^, MD; Weiju Li^10^, MD; Wei Sun^11^, MD; Dong Wu^9^, PhD; Wentian Cao^3^, PhD; Xiaoqin Mai^1^, PhD; Gong Chen^5^, PhD; Dongmin Wang^6^*, PhD; Qihong Zou^2,3^*, PhD

^1^Department of Psychology, Renmin University of China, Beijing, China

^2^Center for MRI Research, Academy for Advanced Interdisciplinary Studies, Peking University, Beijing, China

^3^Beijing City Key Lab for Medical Physics and Engineering, Institution of Heavy Ion

Physics, School of Physics, Peking University, Beijing, China

^4^Center for Magnetic Resonance Imaging Research & Key Laboratory of Applied Brain and Cognitive Sciences, School of Business and Management, Shanghai International Studies University, Shanghai, China

^5^Institute of Population Research, Peking University, Beijing, China

^6^Department of Physical Education, Peking University, Beijing, China

^7^Sports Coaching College, Beijing Sports University, Beijing, China

^8^The School of Sports Science, Beijing Sport University, Beijing, China

^9^China Wushu School, Beijing Sport University, Beijing, China

^10^Peking University Hospital, Beijing, China

^11^National Clinical Research Center for Mental Disorders (Peking University Sixth Hospital), Beijing, China

^*^Correspondence to: Dr. Qihong Zou, Center for MRI Research, Peking University, 5 Yiheyuan Road, Haidian District, Beijing, China 100871, E-mail: [zouqihong@pku.edu.cn](mailto:zouqihong@pku.edu.cn); or Dr. Dongmin Wang, E-Mail: [dongmin_wang@pku.edu.cn](mailto:dongmin_wang@pku.edu.cn).

**SUPPLEMENTARY MATERIAL**

Contents

[**Methods** 3](#_Toc157715345)

[Enrollment and allocation 3](#_Toc157715346)

[Sample size estimation 3](#_Toc157715347)

[Exercise intervention 3](#_Toc157715348)

[**Results** 5](#_Toc157715349)

[Table S1. The exercise intervention protocol 5](#_Toc157715350)

[Table S2. Baseline demographic and clinical characteristics of the insomnia group and healthy controls 11](#_Toc157715351)

[Table S3. Baseline demographic and clinical characteristics of the exercise group and waitlist group 12](#_Toc157715352)

[Table S4. Regions showing significant differences in functional connectivity with the left M1 in insomnia individuals and healthy controls 13](#_Toc157715353)

[Table S5. Regions showing significant differences in functional connectivity with the right M1 in insomnia individuals and healthy controls 14](#_Toc157715354)

[Table S6. Outcomes 15](#_Toc157715355)

[Table S7. PSQI Outcomes 18](#_Toc157715356)

[Table S8. Correlation between changes in functional connectivity and improvements in sleep quality in the exercise group after 12-week exercise program 19](#_Toc157715357)

[Table S9. Correlation between changes in functional connectivity and alleviation in negative emotion in the exercise group after 12-week exercise program 20](#_Toc157715358)

[Fig. S1. Flow of participants through each stage. 1](#_Toc157715359)

## Methods

## Enrollment and allocation

The insomnia individuals sequentially allocated to the 12-week exercise intervention (EX) or the waitlist control condition (WL) according to the order of enrollment, with the first half of participants assigned to the EX group and the other half assigned to the WL group. To ensure the effect of the intervention, each community-based group had no less than 8 and no more than 16 participants, the final number of participants were determined by the number of participants in the community groups.

Considering the potential drop out and withdraw (35%) during the intervention, the minimum sample size at baseline is 40 for each group. We recruited participants from five random chosen neighboring communities. The number of participants in WL group recruited depending on EX group, which is basically the same as that in EX group. After the 12-week intervention, participants with insomnia were invited to come back for sleep evaluation and MRI assessment, with the same data acquisition parameters as at baseline. The number of HCs individuals recruited depending on the numbers of insomnia participants. Baseline data were acquired from HCs after their enrollment.

## Sample size estimation

A power calculation indicated that the total sample size necessary for sufficient statistical power was 128 (64 in each group) for detecting differences on a t test; this calculation was performed G*Power 3.1 with the following parameters: two tailed, effect size *d* =0.5 (medium effect size), power = 0.80, and *α* = 0.05.

As the study employed a two-armed pretest-posttest design, estimation of the sample size was performed using G*Power 3.1 by setting the test family section and statistical test section as “*F* tests” and “ANOVA: Repeated measures, within-between interaction”. Using this setting, an interaction effect size (*d*) = 0.25, 2 groups and 2 measurement time points, the results indicated that 52 participants (26 in each group) were needed to achieve a statistical power of 80% (*α* = 0.01).

## Exercise intervention

*Exercise group*

The exercise intervention protocol was designed by our team, which has a multidisciplinary background and includes clinical doctors, sports researchers, and Tai Chi instructors. Participants in the EX group performed an exercise intervention that includes Tai Chi [warm-up (Tai Chi Chan Si Gong), Zhuang Gong (including Tai Chi Zhuang and Tai Chi Ball), and Chen-style 8-Form Tai Chi] and resistance training.

The Tai Chi instructors were trained and certified by Peking University and Beijing Sports University. Resistance instructors were certified by the Sports Medicine Laboratory, Peking University. Both sets of instructors have at least 5 years of experience teaching older adults. In addition, the instructors in this study completed the required research and human subject protection training before the exercise intervention. Adjustments of intensity, range of motion, and the numbers of repeated exercises have been performed by the instructors to ensure safe practice. All the EX participants engage in four sessions a week, each lasting one hour, in a community setting. The exercise intervention performed by community-based groups and the exercise location was vary flexibly depending on the weather. Compliance and adherence is increased by daily communication via WeChat, feedback of sleep quality and brain imaging at baseline, and delivering mid-term gifts.

In the first four weeks of the intervention, the participants were taught both Chen-style 8-Form Tai Chi and resistance training four times a week. Each session includes 5 min of Tai Chi Chan Si Gong, 5 min of Tai Chi Zhuang, 5 min of Tai Chi Ball, 20 min of learning Tai Chi and resistance training, 20 min of reviewing these exercises, and 5 min of relaxation. In weeks 5 – 12, each session consists of 5 min of Tai Chi Zhuang, 5 min of Tai Chi Chan Si Gong, 5 min of Tai Chi Ball, 40 min of Chen-style 8-Form Tai Chi or resistance training, and 5 min of relaxation.

*Tai Chi Chan Si Gong*

Chan Si Gong is a Chinese approach that aims to cure the “illness of evolution” or “illness of modern society” by enhancing joint flexibility. Chan Si Gong starts with the fingers, passing through the wrists, elbows, shoulders and neck, chest, abdomen, hips, knees, and up to the ankles and toes to relax the whole body. The instructors designed a feasible plan for elderly participants to exercise from fingers to toes, from the articular muscles to the tendons, and from the body to the mind. Chan Si Gong can be used to dredge the meridians and promote circulation; improve joint flexibility and bone strength; build muscles and promote circulation; regulate nerves, and dredge channels and collaterals. In the current study, Tai Chi Chan Si Gong was designed as the warm-up exercise; it includes 9 movements: rubbing the palms and stroking the body (Golden Chicken Shakes Feathers), wrist twisting (Jin Si Chan Wan), stretching elbows (You Long Chu Hai), swiveling elbows (Fan Hua Wu Xiu), bending the upper body (Hai Di Fan Hua), drawing a vertical circle with the hip joint (Niu Zhuan Qian Kun), hip twisting (Dragonfly Briefly Touches the Water), holding the knees and drawing back (Prince Nezha's Triumph Against the Dragon King), turning the ankles and rotating the foot.

*Chen-style 8-form Tai Chi*

Chen-style Tai Chi originated from the village of Chenjiagou in Henan Province, China. This style is characterized by spiral and circle movements, gentle and sustained force, precise movements, slow strength, and internal rotation of Dantian. This exercise is believed to regulate the mind, strengthen the heart and kidneys, increase mental acuity and blood circulation, deepen breathing, benefit the lungs, and promote happiness. Chen-style 8-form Tai Chi includes the following 8 movements: preparation, jin gang dao dui, lan zha yi, liu feng si bi, dan bian, yun shou, dang tou pao, jin gang dao dui, and hou shi.

*Tai Chi Ball*

Tai Chi Ball is one of the basic skills of Tai Chi and a strength training method. The ball can range from 1 kg to 5 kg. Participants should choose an appropriate ball for their physical strength. Tai Chi Ball involves the 8 following movements: preparation, turn body in horse stance with ball, thrust with ball, push ball in bow stance, turn body in bow stance, push ball from waist with single leg, shoulder press with ball with single leg, and lift ball from standing to horse stance.

*Resistance training*

We adopted traditional resistance training combined with Tai Chi elements. Traditional resistance training includes wide air squats, air squat with legs shoulder-width apart, press thruster, hard pull with resistance band, stiff-legged deadlift, side lateral, walking lunge and knees-ups.

*Waitlist control condition*

The WL group maintained their normal activities in the 12-week period. After finishing all assessments, the WL group received a video about the 12-week exercise intervention protocol to improve insomnia through self-practice.

## Results

## Table S1. The exercise intervention protocol

| Session | Contents |
| --- | --- |
| 1 | - 3 min of instruction on Tai Chi’s history, function, and basic working principle - 5 min of Tai Chi Chan Si Gong - 5 min of Tai Chi Ball - 5 min of Tai Chi Zhuang - 20 min of learning Chen-style 8-form Tai Chi - 17 min of reviewing Chen-style 8-form Tai Chi - 5 min of relaxation |
| 2 | - 5 min of Tai Chi Chan Si Gong - 5 min of Tai Chi Ball - 5 min of Tai Chi Zhuang - 20 min of learning Chen-style 8-form Tai Chi - 20 min of reviewing Chen-style 8-form Tai Chi - 5 min of relaxation |
| 3 | - 5 min of Tai Chi Chan Si Gong - 5 min of Tai Chi Ball - 5 min of Tai Chi Zhuang - 40 min of resistance training - 5 min of relaxation |
| 4 | - 5 min of Tai Chi Chan Si Gong - 5 min of Tai Chi Ball - 5 min of Tai Chi Zhuang - 40 min of resistance training - 5 min of relaxation |
| 5 | - 5 min of Tai Chi Chan Si Gong - 5 min of Tai Chi Ball - 5 min of Tai Chi Zhuang - 20 min of learning Chen-style 8-form Tai Chi - 20 min of reviewing Chen-style 8-form Tai Chi - 5 min of relaxation |
| 6 | - 5 min of Tai Chi Chan Si Gong - 5 min of Tai Chi Ball - 5 min of Tai Chi Zhuang - 20 min of learning Chen-style 8-form Tai Chi - 20 min of reviewing Chen-style 8-form Tai Chi - 5 min of relaxation |
| 7 | - 5 min of Tai Chi Chan Si Gong - 5 min of Tai Chi Ball - 5 min of Tai Chi Zhuang - 40 min of resistance training - 5 min of relaxation |

| 8 | - 5 min of Tai Chi Chan Si Gong - 5 min of Tai Chi Ball - 5 min of Tai Chi Zhuang - 40 min of resistance training - 5 min of relaxation |
| --- | --- |
| 9 | - 5 min of Tai Chi Chan Si Gong - 5 min of Tai Chi Ball - 5 min of Tai Chi Zhuang - 20 min of learning Chen-style 8-form Tai Chi - 20 min of reviewing Chen-style 8-form Tai Chi - 5 min of relaxation |
| 10 | - 5 min of Tai Chi Chan Si Gong - 5 min of Tai Chi Ball - 5 min of Tai Chi Zhuang - 20 min of learning Chen-style 8-form Tai Chi - 20 min of reviewing Chen-style 8-form Tai Chi - 5 min of relaxation |
| 11 | - 5 min of Tai Chi Chan Si Gong - 5 min of Tai Chi Ball - 5 min of Tai Chi Zhuang - 40 min of resistance training - 5 min of relaxation |
| 12 | - 5 min of Tai Chi Chan Si Gong - 5 min of Tai Chi Ball - 5 min of Tai Chi Zhuang - 40 min of resistance training - 5 min of relaxation |
| 13 | - 5 min of Tai Chi Chan Si Gong - 5 min of Tai Chi Ball - 5 min of Tai Chi Zhuang - 20 min of learning Chen-style 8-form Tai Chi - 20 min of reviewing Chen-style 8-form Tai Chi - 5 min of relaxation |
| 14 | - 5 min of Tai Chi Chan Si Gong - 5 min of Tai Chi Ball - 5 min of Tai Chi Zhuang - 20 min of learning Chen-style 8-form Tai Chi - 20 min of reviewing Chen-style 8-form Tai Chi - 5 min of relaxation |
| 15 | - 5 min of Tai Chi Chan Si Gong - 5 min of Tai Chi Ball - 5 min of Tai Chi Zhuang - 40 min of resistance training - 5 min of relaxation |
| 16 | - 5 min of Tai Chi Chan Si Gong - 5 min of Tai Chi Ball - 5 min of Tai Chi Zhuang - 40 min of resistance training - 5 min of relaxation |
| 17 | - 5 min of Tai Chi Chan Si Gong - 5 min of Tai Chi Ball - 5 min of Tai Chi Zhuang - 40 min of practicing Chen-style 8-form Tai Chi - 5 min of relaxation |
| 18 | - 5 min of Tai Chi Chan Si Gong - 5 min of Tai Chi Ball - 5 min of Tai Chi Zhuang - 40 min of practicing Chen-style 8-form Tai Chi - 5 min of relaxation |
| 19 | - 5 min of Tai Chi Chan Si Gong - 5 min of Tai Chi Ball - 5 min of Tai Chi Zhuang - 40 min of resistance training - 5 min of relaxation |
| 20 | - 5 min of Tai Chi Chan Si Gong - 5 min of Tai Chi Ball - 5 min of Tai Chi Zhuang - 40 min of resistance training - 5 min of relaxation |
| 21 | - 5 min of Tai Chi Chan Si Gong - 5 min of Tai Chi Ball - 5 min of Tai Chi Zhuang - 40 min of practicing Chen-style 8-form Tai Chi - 5 min of relaxation |
| 22 | - 5 min of Tai Chi Chan Si Gong - 5 min of Tai Chi Ball - 5 min of Tai Chi Zhuang - 40 min of practicing Chen-style 8-form Tai Chi - 5 min of relaxation |
| 23 | - 5 min of Tai Chi Chan Si Gong - 5 min of Tai Chi Ball - 5 min of Tai Chi Zhuang - 40 min of resistance training - 5 min of relaxation |
| 24 | - 5 min of Tai Chi Chan Si Gong - 5 min of Tai Chi Ball - 5 min of Tai Chi Zhuang - 40 min of resistance training - 5 min of relaxation |
| 25 | - 5 min of Tai Chi Chan Si Gong - 5 min of Tai Chi Ball - 5 min of Tai Chi Zhuang - 40 min of practicing Chen-style 8-form Tai Chi - 5 min of relaxation |
| 26 | - 5 min of Tai Chi Chan Si Gong - 5 min of Tai Chi Ball - 5 min of Tai Chi Zhuang - 40 min of practicing Chen-style 8-form Tai Chi - 5 min of relaxation |
| 27 | - 5 min of Tai Chi Chan Si Gong - 5 min of Tai Chi Ball - 5 min of Tai Chi Zhuang - 40 min of resistance training - 5 min of relaxation |
| 28 | - 5 min of Tai Chi Chan Si Gong - 5 min of Tai Chi Ball - 5 min of Tai Chi Zhuang - 40 min of resistance training - 5 min of relaxation |
| 29 | - 5 min of Tai Chi Chan Si Gong - 5 min of Tai Chi Ball - 5 min of Tai Chi Zhuang - 40 min of practicing Chen-style 8-form Tai Chi - 5 min of relaxation |
| 30 | - 5 min of Tai Chi Chan Si Gong - 5 min of Tai Chi Ball - 5 min of Tai Chi Zhuang - 40 min of practicing Chen-style 8-form Tai Chi - 5 min of relaxation |
| 31 | - 5 min of Tai Chi Chan Si Gong - 5 min of Tai Chi Ball - 5 min of Tai Chi Zhuang - 40 min of resistance training - 5 min of relaxation |
| 32 | - 5 min of Tai Chi Chan Si Gong - 5 min of Tai Chi Ball - 5 min of Tai Chi Zhuang - 40 min of resistance training - 5 min of relaxation |
| 33 | - 5 min of Tai Chi Chan Si Gong - 5 min of Tai Chi Ball - 5 min of Tai Chi Zhuang - 40 min of practicing Chen-style 8-form Tai Chi - 5 min of relaxation |
| 34 | - 5 min of Tai Chi Chan Si Gong - 5 min of Tai Chi Ball - 5 min of Tai Chi Zhuang - 40 min of practicing Chen-style 8-form Tai Chi - 5 min of relaxation |
| 35 | - 5 min of Tai Chi Chan Si Gong - 5 min of Tai Chi Ball - 5 min of Tai Chi Zhuang - 40 min of resistance training - 5 min of relaxation |
| 36 | - 5 min of Tai Chi Chan Si Gong - 5 min of Tai Chi Ball - 5 min of Tai Chi Zhuang - 40 min of resistance training - 5 min of relaxation |
| 37 | - 5 min of Tai Chi Chan Si Gong - 5 min of Tai Chi Ball - 5 min of Tai Chi Zhuang - 40 min of practicing Chen-style 8-form Tai Chi - 5 min of relaxation |
| 38 | - 5 min of Tai Chi Chan Si Gong - 5 min of Tai Chi Ball - 5 min of Tai Chi Zhuang - 40 min of practicing Chen-style 8-form Tai Chi - 5 min of relaxation |
| 39 | - 5 min of Tai Chi Chan Si Gong - 5 min of Tai Chi Ball - 5 min of Tai Chi Zhuang - 40 min of resistance training - 5 min of relaxation |
| 40 | - 5 min of Tai Chi Chan Si Gong - 5 min of Tai Chi Ball - 5 min of Tai Chi Zhuang - 40 min of resistance training - 5 min of relaxation |
| 41 | - 5 min of Tai Chi Chan Si Gong - 5 min of Tai Chi Ball - 5 min of Tai Chi Zhuang - 40 min of practicing Chen-style 8-form Tai Chi - 5 min of relaxation |
| 42 | - 5 min of Tai Chi Chan Si Gong - 5 min of Tai Chi Ball - 5 min of Tai Chi Zhuang - 40 min of practicing Chen-style 8-form Tai Chi - 5 min of relaxation |
| 43 | - 5 min of Tai Chi Chan Si Gong - 5 min of Tai Chi Ball - 5 min of Tai Chi Zhuang - 40 min of resistance training - 5 min of relaxation |
| 44 | - 5 min of Tai Chi Chan Si Gong - 5 min of Tai Chi Ball - 5 min of Tai Chi Zhuang - 40 min of resistance training - 5 min of relaxation |
| 45 | - 5 min of Tai Chi Chan Si Gong - 5 min of Tai Chi Ball - 5 min of Tai Chi Zhuang - 40 min of practicing Chen-style 8-form Tai Chi - 5 min of relaxation |
| 46 | - 5 min of Tai Chi Chan Si Gong - 5 min of Tai Chi Ball - 5 min of Tai Chi Zhuang - 40 min of practicing Chen-style 8-form Tai Chi - 5 min of relaxation |
| 47 | - 5 min of Tai Chi Chan Si Gong - 5 min of Tai Chi Ball - 5 min of Tai Chi Zhuang - 40 min of resistance training - 5 min of relaxation |
| 48 | - 5 min of Tai Chi Chan Si Gong - 5 min of Tai Chi Ball - 5 min of Tai Chi Zhuang - 40 min of resistance training - 5 min of relaxation |

The training sessions were run by certified fitness instructors/Tai Chi masters with at least 5 years of experience teaching older adults. The performance of the participants was continuously and closely monitored by the instructors. Adjustments of intensity, range of motion and numbers of repeated exercises were made when deemed necessary by the instructors to ensure the safe practice of the exercise program.

| Table S2. Baseline demographic and clinical characteristics of the insomnia group and healthy controls | | | | | | | | |  |
| --- | --- | --- | --- | --- | --- | --- | --- | --- | --- |
|  | **Insomnia group**  **(n = 85)** | | **Healthy controls**  **(n = 84)** | | ***P* value^a^** | | |  |  |
| ***Baseline demographic and clinical characteristics*** | | | | | | | | |  |
| Age, years, mean (SD) | | 62.27 (6.18) | | 62.80 (6.40) | | 0.587 | | |  |
| Sex, female, n (%) | | 82.35 | | 80.95 | | 0.814 | | |  |
| Years of education, years, mean (SD) | | 12.01 (2.87) | | 12.68 (2.99) | | 0.135 | | |  |
| MMSE score, mean (SD) | | 28.79 (1.13) | | 28.86 (1.26) | | 0.709 | | |  |
| BMI, kg/m^2^, mean (SD) | | 23.93 (2.72) | | 23.51 (2.22) | | 0.272 | | |  |
| IPAQ, MET, mean (SD) | | 6467.12 (4223.90) | | 6450.02 (2372.34) | | 0.974 | | |  |
| History of drug use, n (%) | | 0 (0) | | 0 (0) | | - | | |  |
| History of psychiatric or neurological illness, n (%) | | 0 (0) | | 0 (0) | | - | | |  |
| ***Self-reported sleep quality and negative emotion*** | | | | | | | | |  |
| ISI score, mean (SD) | | 15.52 (4.10) | | 1.35 (1.59) | | | < 0.001 | | |
| PSQI score, mean (SD) | | 11.82 (3.25) | | 3.60 (1.95) | | | < 0.001 | | |
| SAS score, mean (SD) | | 46.93 (8.38) | | 35.58 (7.63) | | | < 0.001 | | |
| SDS score, mean (SD) | | 55.41 (10.06) | | 40.80 (11.01) | | | < 0.001 | | |
| ***Objective sleep quality*** | | | | | | | | |  |
| Sleep duration, min, mean (SD) ^b^ | | 354.84 (60.30) | | 355.27 (54.92) | | | 0.963 | | |
| Light sleep duration, min, mean (SD) ^b^ | | 247.96 (46.95) | | 248.50 (49.22) | | | 0.944 | | |
| Deep sleep duration, min, mean (SD) ^b^ | | 56.07 (24.11) | | 56.58 (23.93) | | | 0.895 | | |
| REM sleep duration, min, mean (SD) ^b^ | | 50.81 (25.67) | | 50.19(23.94) | | | 0.875 | | |
| Number of awakenings, mean (SD) ^b^ | | 19.10 (8.76) | | 18.24 (7.45) | | | 0.507 | | |
| WASO, min, mean (SD) ^b^ | | 76.55 (49.89) | | 51.03 (27.91) | | | < 0.001 | | |
| RSOL, min, mean (SD) ^c^ | | 133.24 (81.17) | | 110.16 (60.80) | | | 0.048 | | |
| ^a^*P* values of group difference were obtained using independent-sample *t* test or chi-squared (χ^2^) test for sex. ^b^Data from 79 insomnia individuals and 79 healthy controls. ^c^Data from 78 insomnia individuals and 76 healthy controls.  SD, standard deviation; MMSE, Mini-Mental State Examination; BMI, Body Mass Index; IPAQ, International Physical Activity Questionnaire; ISI, Insomnia Severity Index; PSQI, Pittsburgh Sleep Quality Index; SAS, Self-Rating Anxiety Scale; SDS, Self-Rating Depression Scale; REM, rapid eye movement; WASO, wakefulness after sleep onset; RSOL, rapid eye movement sleep onset latency. | | | | | | | | |  |

| Table S3. Baseline demographic and clinical characteristics of the exercise group and waitlist group | | | |  |  |
| --- | --- | --- | --- | --- | --- |
|  | **Exercise**  **(n = 37)** | **Waitlist**  **(n = 30)** | ***P* value^a^** | |  |
| ***Baseline demographic and clinical characteristics*** | | | | | |
| Age, years, mean (SD) | 61.62 (5.97) | 61.90 (6.40) | 0.855 | |  |
| Sex, female, n (%) | 89.19 | 73.33 | 0.092 | |  |
| Years of education, mean (SD) | 11.93 (2.98) | 11.92 (2.86) | 0.983 | |  |
| MMSE score, mean (SD) | 28.76 (1.01) | 28.80 (1.19) | 0.873 | |  |
| BMI, kg/m^2^, mean (SD) | 23.78 (2.72) | 24.15 (2.66) | 0.587 | |  |
| IPAQ, MET, mean (SD) | 7095.90 (5256.07) | 6125.95 (3010.82) | 0.373 | |  |
| History of drug use, n (%) | 0 (0) | 0 (0) | - | |  |
| History of psychiatric or neurological illness, n (%) | 0 (0) | 0 (0) | - | |  |
| ***Self-reported sleep quality and negative emotion*** | | | | | |
| ISI score, mean (SD) | 15.16 (4.48) | 15.60 (3.53) | 0.664 | |  |
| PSQI score, mean (SD) | 11.84 (3.40) | 11.73 (3.13) | 0.897 | |  |
| SAS score, mean (SD) | 48.43 (8.28) | 46.97 (9.00) | 0.491 | |  |
| SDS score, mean (SD) | 59.24 (7.29) | 50.33 (10.59) | < 0.001 | |  |
| ***Objective sleep quality*** | | | | |  |
| Sleep duration, min, mean (SD) ^b^ | 354.14 (54.37) | 363.12 (72.03) | 0.565 | |  |
| Light sleep duration, min, mean (SD) ^b^ | 244.31 (45.29) | 258.91 (52.62) | 0.230 | |  |
| Deep sleep duration, min, mean (SD) ^b^ | 59.24 (24.44) | 50.84 (24.56) | 0.172 | |  |
| REM sleep duration, min, mean (SD) ^b^ | 50.58 (26.13) | 53.36 (28.03) | 0.679 | |  |
| Number of awakenings, mean (SD) ^b^ | 18.65 (8.35) | 19.72 (8.08) | 0.600 | |  |
| WASO, min, mean (SD) ^b^ | 81.85 (52.54) | 63.98 (31.60) | 0.092 | |  |
| RSOL, min, mean (SD) ^c^ | 127.17 (60.25) | 121.91 (88.00) | 0.777 | |  |
| ^a^*P* values for group differences were obtained using independent-sample *t* tests or chi-squared (χ^2^) tests for sex. ^b^Data from 37 participants in the exercise group and 29 participants in the waitlist group. ^c^Data from 36 participants in the exercise group and 29 participants in the waitlist group.  SD, standard deviation; MMSE, Mini-Mental State Examination; BMI, Body Mass Index; IPAQ, International Physical Activity Questionnaire; ISI, Insomnia Severity Index; PSQI, Pittsburgh Sleep Quality Index; SAS, Self-Rating Anxiety Scale; SDS, Self-Rating Depression Scale; REM, rapid eye movement; WASO, wakefulness after sleep onset; RSOL, rapid eye movement sleep onset latency. | | | | | |

| Table S4. Regions showing significant differences in functional connectivity with the left M1 in insomnia individuals and healthy controls | | | | | | |
| --- | --- | --- | --- | --- | --- | --- |
| **Region** | **Hemisphere** | **Number of voxels** | **Peak *t***  **value** | **Peak coordinates** | | |
|  |  |  |  | **x** | **y** | **z** |
| ***Insomnia < HC*** |  |  |  |  |  |  |
| M1/S1 | L | 1198 | -4.87 | -44 | -22 | 66 |
| SMA/M1/S1 | R | 865 | -5.09 | 12 | -20 | 56 |
| M1/S1 | R | 506 | -4.66 | 62 | 2 | 28 |
| SMA | L | 322 | -4.48 | -8 | -16 | 70 |
| ***Insomnia > HC*** |  |  |  |  |  |  |
| CAU/PUT/THA | R | 697 | 4.52 | 22 | -4 | 20 |
| CEB | R | 605 | 4.52 | 14 | -78 | -28 |
| SPL | L | 559 | 4.68 | -54 | -52 | 50 |
| CAU/PUT/THA | L | 511 | 4.69 | -22 | 12 | 14 |
| PCC | M | 435 | 4.49 | 4 | -40 | 24 |
| CEB | L | 363 | 4.12 | -12 | -84 | -26 |
| M1, primary motor cortex; S1, primary sensory cortex; SMA, supplementary motor area; CAU, caudate; PUT, putamen; THA, thalamus; CEB, cerebellum; SPL, superior parietal lobule; PCC, posterior cingulate cortex; R, right; L, left; M, medial. The voxel size was 8 mm^3^, and the coordinates are presented in Montreal Neurological Institute (MNI) space. | | | | | | |

| Table S5. Regions showing significant differences in functional connectivity with the right M1 in insomnia individuals and healthy controls | | | | | | |
| --- | --- | --- | --- | --- | --- | --- |
| **Region** | **Hemisphere** | **Number of voxels** | **Peak *t***  **value** | **Peak coordinates** | | |
|  |  |  |  | **x** | **y** | **z** |
| ***Insomnia < HC*** |  |  |  |  |  |  |
| M1/SMA | L | 2656 | -6.05 | -10 | -22 | 52 |
| M1/SMA | R | 1049 | -4.76 | 22 | -22 | 56 |
| M1/S1 | R | 527 | -4.50 | 54 | -6 | 20 |
| ***Insomnia > HC*** |  |  |  |  |  |  |
| SPL | L | 642 | 5.16 | -52 | -52 | 48 |
| CAU/PUT | R | 561 | 4.97 | 22 | 18 | 12 |
| PCC | M | 249 | 4.41 | 4 | -36 | 26 |
| HC, healthy control; M1, primary motor cortex; SMA, supplementary motor area; S1, primary sensory cortex; SPL, superior parietal lobule; CAU, caudate; PUT, putamen; PCC, posterior cingulate cortex; L, left; R, right; M, medial. The voxel size was 8 mm^3^, and the coordinates are presented in MNI space. | | | | | | |

| Table S6. Outcomes | | | | | | | | | | | | |
| --- | --- | --- | --- | --- | --- | --- | --- | --- | --- | --- | --- | --- |
| Measurement | Mean (SD) | |  | *P* value^a^ | | | | | | | | |
|  | Baseline | Week 12 |  | Interaction effect | | | | Group effect | | | | Time effect |
| **Primary outcomes** | | | | | | | | | | | | |
| ***Self-reported sleep quality and negative emotion*** | | | | | | | | | | | | |
| **ISI score** ^b^ | | | | | | | | | | | | |
| Exercise | 15.16 (4.48) | 10.97 (4.00) |  | 0.008 | | | | 0.033 | | | | < 0.001 |
| Waitlist | 15.60 (3.53) | 14.33 (5.09) |  |  |  |  |  |  |  |  |  |  |
| **PSQI score** ^b^ | | | | | | | | | | | | |
| Exercise | 11.84 (3.40) | 8.35 (3.85) |  | < 0.001 | | | | 0.039 | | | | < 0.001 |
| Waitlist | 11.73 (3.13) | 11.67 (3.79) |  |  |  |  |  |  |  |  |  |  |
| **SAS score** ^b^ | | | | | | | | | | | | |
| Exercise | 48.43 (8.28) | 42.46 (8.55) |  | 0.010 | | | 0.489 | | | | | 0.002 |
| Waitlist | 46.97 (9.00) | 46.43 (8.70) |  |  |  |  |  |  |  |  |  |  |
| **SDS score** ^b^ | | | | | | | | | | | | |
| Exercise | 59.24 (7.29) | 47.19 (13.81) |  | < 0.001 | | | 0.304 | | | < 0.001 | | |
| Waitlist | 50.33 (10.59) | 51.80 (10.51) |  |  |  |  |  |  |  |  |  |  |
| ***Objective sleep quality*** | | | | | | | | | | | | |
| **Sleep duration, min** ^c^ | | | | | | | | | | | | |
| Exercise | 354.14 (54.37) | 364.01 (45.06) |  | 0.689 | | | 0.639 | | | 0.391 | | |
| Waitlist | 363.12 (72.03) | 366.71 (62.43) |  |  |  |  |  |  |  |  |  |  |
| **Light sleep duration, min** ^c^ | | | | | | | | | | | | |
| Exercise | 244.31 (45.29) | 257.62 (46.85) |  | 0.572 | | | | 0.304 | | | | 0.225 |
| Waitlist | 258.91 (52.62) | 263.78 (57.94) |  |  |  |  |  |  |  |  |  |  |
| **Deep sleep duration, min** ^c^ | | | | | | | | | | | | |
| Exercise | 59.24 (24.44) | 52.59 (24.31) |  | 0.153 | | | | 0.375 | | | | 0.260 |
| Waitlist | 50.84 (24.56) | 51.64 (23.05) |  |  |  |  |  |  |  |  |  |  |
| **REM sleep duration, min** ^c^ | | | | | | | | | | | | |
| Exercise | 50.58 (26.13) | 53.80 (22.51) |  | | | 0.302 | | | 0.981 | | | 0.823 |
| Waitlist | 53.36 (28.03) | 51.29 (26.67) |  | | |  |  |  |  |  |  |  |
| **Number of awakenings, times** ^c^ | | | | | | | | | | | | |
| Exercise | 18.65 (8.35) | 16.86 (6.72) |  | | | 0.460 | | | 0.326 | | | 0.236 |
| Waitlist | 19.72 (8.08) | 19.31 (9.42) |  | | |  |  |  |  |  |  |  |
| **WASO, min** ^c^ | | | | | | | | | | | | |
| Exercise | 81.85 (52.54) | 58.77 (40.32) |  | | 0.073 | | | 0.363 | | | 0.044 | |
| Waitlist | 63.98 (31.60) | 62.64 (35.95) |  | |  |  |  |  |  |  |  |  |
| **RSOL, min** ^d^ | | | | | | | | | | | | |
| Exercise | 127.17 (60.25) | 119.06 (68.15) |  | 0.977 | | | 0.654 | | | | 0.513 | |
| Waitlist | 121.91 (88.00) | 113.07 (71.43) |  |  |  |  |  |  |  |  |  |  |
| **Secondary outcomes** | | | | | | | | | | | | |
| ***Functional connectivity of the left M1*** | | | | | | | | | | | | |
| **M1/S1_L** ^b^ | | | | | | | | | | | | |
| Exercise | 0.64 (0.16) | 0.70 (0.14) |  | 0.980 | | | 0.251 | | | | 0.031 | |
| Waitlist | 0.68 (0.16) | 0.74 (0.22) |  |  |  |  |  |  |  |  |  |  |
| **SMA/M1/S1_R** ^b^ | | | | | | | | | | | | |
| Exercise | 0.30 (0.15) | 0.34 (0.15) |  | 0.882 | | | | 0.804 | | | 0.109 | |
| Waitlist | 0.31 (0.14) | 0.35 (0.18) |  |  |  |  |  |  |  |  |  |  |
| **CAU/PUT/THA_R** ^b^ | | | | | | | | | | | | |
| Exercise | 0.02 (0.16) | -0.09 (0.19) |  | 0.364 | | | | 0.715 | | | | 0.004 |
| Waitlist | 0.01 (0.21) | -0.05 (0.22) |  |  |  |  |  |  |  |  |  |  |
| **CEB_R** ^b^ | | | | | | | | | | | | |
| Exercise | -0.11 (0.15) | -0.25 (0.15) |  | 0.003 | | | | 0.866 | | | | 0.001 |
| Waitlist | -0.17 (0.13) | -0.18 (0.20) |  |  |  |  |  |  |  |  |  |  |
| **SPL_L** ^b^ | | | | | | | | | | | | |
| Exercise | -0.08 (0.16) | -0.13 (0.19) |  | 0.968 | | | 0.896 | | | | | 0.017 |
| Waitlist | -0.07 (0.17) | -0.13 (0.17) |  |  |  |  |  |  |  |  |  |  |
| **CAU/PUT/THA_L** ^b^ | | | | | | | | | | | | |
| Exercise | 0.07 (0.17) | -0.05 (0.19) |  | 0.379 | | | 0.704 | | | 0.001 | | |
| Waitlist | 0.06 (0.20) | -0.01 (0.21) |  |  |  |  |  |  |  |  |  |  |
| **M1/S1_R** ^b^ | | | | | | | | | | | | |
| Exercise | 0.53 (0.20) | 0.58 (0.24) |  | 0.973 | | | 0.667 | | | 0.138 | | |
| Waitlist | 0.51 (0.20) | 0.56 (0.23) |  |  |  |  |  |  |  |  |  |  |
| **PCC_M** ^b^ | | | | | | | | | | | | |
| Exercise | -0.19 (0.16) | -0.19 (0.19) |  | 0.863 | | | | 0.213 | | | | 0.859 |
| Waitlist | -0.14 (0.15) | -0.15 (0.18) |  |  |  |  |  |  |  |  |  |  |
| **CEB_L** ^b^ | | | | | | | | | | | | |
| Exercise | -0.20 (0.17) | -0.33 (0.14) |  | 0.011 | | | | 0.936 | | | | < 0.001 |
| Waitlist | -0.25 (0.14) | -0.27 (0.18) |  |  |  |  |  |  |  |  |  |  |
| **SMA_L** ^b^ | | | | | | | | | | | | |
| Exercise | 0.34 (0.14) | 0.40 (0.19) |  | | | 0.569 | | | 0.572 | | | 0.085 |
| Waitlist | 0.37 (0.17) | 0.40 (0.18) |  | | |  |  |  |  |  |  |  |
| ***Functional connectivity of the right M1*** | | | | | | | | | | | | |
| **M1/SMA_L** ^b^ | | | | | | | | | | | | |
| Exercise | 0.44 (0.18) | 0.49 (0.20) |  | | | 0.472 | | | 0.727 | | | 0.169 |
| Waitlist | 0.47 (0.18) | 0.49 (0.23) |  | | |  |  |  |  |  |  |  |
| **M1/SMA_R** ^b^ | | | | | | | | | | | | |
| Exercise | 0.54 (0.18) | 0.63 (0.20) |  | | 0.482 | | | 0.781 | | | 0.007 | |
| Waitlist | 0.55 (0.15) | 0.60 (0.21) |  | |  |  |  |  |  |  |  |  |
| **SPL_L** ^b^ | | | | | | | | | | | | |
| Exercise | -0.08 (0.15) | -0.15 (0.17) |  | 0.856 | | | 0.554 | | | | 0.004 | |
| Waitlist | -0.10 (0.17) | -0.16 (0.18) |  |  |  |  |  |  |  |  |  |  |
| **CAU/PUT_R** ^b^ | | | | | | | | | | | | |
| Exercise | 0.02 (0.17) | -0.06 (0.21) |  | 0.538 | | | 0.882 | | | | 0.012 | |
| Waitlist | 0.00 (0.20) | 0.05 (0.21) |  |  |  |  |  |  |  |  |  |  |
| **M1/S1_R** ^b^ | | | | | | | | | | | | |
| Exercise | 0.48 (0.21) | 0.54 (0.22) |  | 0.393 | | | 0.509 | | | | 0.334 | |
| Waitlist | 0.48 (0.19) | 0.48 (0.25) |  |  |  |  |  |  |  |  |  |  |
| **PCC_M** ^b^ | | | | | | | | | | | | |
| Exercise | -0.18 (0.15) | -0.19 (0.20) |  | 0.812 | | | 0.268 | | | | 0.973 | |
| Waitlist | -0.15 (0.18) | -0.14 (0.20) |  |  |  |  |  |  |  |  |  |  |

^a^*P* values for the interaction effect, group effect and time effect were obtained using a generalized estimated equation (GEE) model. ^b^Data from 37 participants in the exercise group and 30 participants in the waitlist group. ^c^Data from 37 participants in the exercise group and 29 participants in the waitlist group. ^d^Data from 36 participants in the exercise group and 29 participants in the waitlist group.

ISI, Insomnia Severity Index; PSQI, Pittsburgh Sleep Quality Index; SAS, Self-Rating Anxiety Scale; SDS, Self-Rating Depression Scale; WASO, wakefulness after sleep onset. REM, rapid eye movement; RSOL, rapid eye movement sleep onset latency; M1, primary motor cortex; S1, primary sensory cortex; SMA, supplementary motor area; CAU, caudate; PUT, putamen; THA, thalamus; CEB, cerebellum; SPL, superior parietal lobule; PCC, posterior cingulate cortex; L, left; R, right; M, medial.

| **Table S7. PSQI Outcomes** | | | | | | |
| --- | --- | --- | --- | --- | --- | --- |
| Measurement | Mean (SD) | |  | *P* value^a^ | | |
|  | Baseline | Week 12 |  | Interaction effect | Group effect | Time effect |
| **Sleep quality score** | | | | | | |
| Exercise | 1.97 (0.55) | 1.32 (0.85) |  | < 0.001 | 0.016 | < 0.001 |
| Waitlist | 2.00 (0.46) | 1.93 (0.69) |  |  |  |  |
| **Sleep latency score** | | | | | | |
| Exercise | 2.03 (0.90) | 1.46 (0.80) |  | 0.044 | 0.351 | 0.001 |
| Waitlist | 2.00 (0.95) | 1.87 (1.11) |  |  |  |  |
| **Sleep duration score** | | | | | | |
| Exercise | 2.27 (0.77) | 1.89 (0.84) |  | 0.052 | 0.026 | 0.104 |
| Waitlist | 2.40 (0.62) | 2.43 (0.82) |  |  |  |  |
| **Habitual sleep efficiency score** | | | | | | |
| Exercise | 1.65 (1.09) | 0.73 (0.84) |  | < 0.001 | 0.052 | 0.028 |
| Waitlist | 1.47(1.22) | 1.83 (1.21) |  |  |  |  |
| **Sleep disturbances score** | | | | | | |
| Exercise | 1.38 (0.64) | 1.14 (0.42) |  | 0.427 | 0.188 | 0.006 |
| Waitlist | 1.47 (0.51) | 1.33 (0.55) |  |  |  |  |
| **Use of sleep medication score** | | | | | | |
| Exercise | 0.65 (1.03) | 0.51 (0.99) |  | 0.679 | 0.541 | 0.222 |
| Waitlist | 0.77 (1.10) | 0.70 (1.18) |  |  |  |  |
| **Daytime dysfunction score** | | | | | | |
| Exercise | 1.89 (0.81) | 1.30 (0.91) |  | 0.004 | 0.868 | 0.004 |
| Waitlist | 1.57 (0.82) | 1.57 (0.73) |  |  |  |  |

^a^*P* values for the interaction effect, group effect and time effect were obtained using a generalized estimated equation (GEE) model.

## Table S8. Correlation between changes in functional connectivity and improvements in sleep quality in the exercise group after 12-week exercise program

| **Regions of interest** | **ISI** | | **PSQI** | |
| --- | --- | --- | --- | --- |
|  | *R value* | *P value* | *R value* | *P value* |
| ***Functional connectivity of the left M1*** | | | | |
| **M1/S1_L** | -0.50 | 0.002 | -0.29 | 0.080 |
| **SMA/M1/S1_R** | -0.34 | 0.039 | -0.34 | 0.037 |
| **CAU/PUT/THA_R** | 0.24 | 0.150 | 0.10 | 0.550 |
| **CEB_R** | 0.07 | 0.694 | -0.12 | 0.497 |
| **SPL_L** | 0.27 | 0.106 | -0.25 | 0.128 |
| **CAU/PUT/THA_L** | 0.19 | 0.264 | 0.06 | 0.735 |
| **M1/S1_R** | -0.09 | 0.576 | 0.21 | 0.222 |
| **PCC_M** | 0.13 | 0.436 | 0.10 | 0.551 |
| **CEB_L** | 0.17 | 0.324 | 0.01 | 0.948 |
| **SMA_L** | -0.45 | 0.006 | -0.33 | 0.046 |
| ***Functional connectivity of the right M1*** | | | | |
| **M1/SMA_L** | -0.48 | 0.003 | -0.16 | 0.332 |
| **M1/SMA_R** | -0.37 | 0.023 | -0.27 | 0.100 |
| **SPL_L** | 0.41 | 0.011 | 0.04 | 0.796 |
| **CAU/PUT_R** | 0.17 | 0.321 | 0.01 | 0.962 |
| **M1/S1_R** | -0.12 | 0.492 | 0.01 | 0.950 |
| **PCC_M** | -0.03 | 0.870 | -0.08 | 0.630 |

ISI, Insomnia Severity Index; PSQI, Pittsburgh Sleep Quality Index; M1, primary motor cortex; S1, primary sensory cortex; SMA, supplementary motor area; CAU, caudate; PUT, putamen; THA, thalamus; CEB, cerebellum; SPL, superior parietal lobule; PCC, posterior cingulate cortex; L, left; R, right; M, medial.

## Table S9. Correlation between changes in functional connectivity and alleviation in negative emotion in the exercise group after 12-week exercise program

| **Regions of interest** | **SAS** | | **SDS** | |
| --- | --- | --- | --- | --- |
|  | *R value* | *P value* | *R value* | *P value* |
| ***Functional connectivity of the left M1*** | | | | |
| **M1/S1_L** | -0.38 | 0.019 | 0.18 | 0.281 |
| **SMA/M1/S1_R** | -0.31 | 0.058 | -0.06 | 0.717 |
| **CAU/PUT/THA_R** | 0.35 | 0.035 | -0.23 | 0.175 |
| **CEB_R** | 0.13 | 0.428 | -0.10 | 0.537 |
| **SPL_L** | 0.05 | 0.777 | -0.11 | 0.513 |
| **CAU/PUT/THA_L** | 0.33 | 0.046 | -0.11 | 0.499 |
| **M1/S1_R** | -0.06 | 0.738 | -0.04 | 0.797 |
| **PCC_M** | 0.15 | 0.386 | 0.08 | 0.624 |
| **CEB_L** | 0.21 | 0.210 | -0.05 | 0.756 |
| **SMA_L** | -0.35 | 0.035 | 0.02 | 0.915 |
| ***Functional connectivity of the right M1*** | | | | |
| **M1/SMA_L** | -0.33 | 0.045 | 0.03 | 0.880 |
| **M1/SMA_R** | -0.40 | 0.014 | -0.01 | 0.958 |
| **SPL_L** | 0.18 | 0.289 | -0.09 | 0.582 |
| **CAU/PUT_R** | 0.24 | 0.148 | -0.04 | 0.791 |
| **M1/S1_R** | -0.22 | 0.197 | 0.03 | 0.875 |
| **PCC_M** | 0.03 | 0.859 | -0.07 | 0.700 |

SAS, Self-Rating Anxiety Scale; SDS, Self-Rating Depression Scale; M1, primary motor cortex; S1, primary sensory cortex; SMA, supplementary motor area; CAU, caudate; PUT, putamen; THA, thalamus; CEB, cerebellum; SPL, superior parietal lobule; PCC, posterior cingulate cortex; L, left; R, right; M, medial.

## Fig. S1. Flow of participants through each stage.


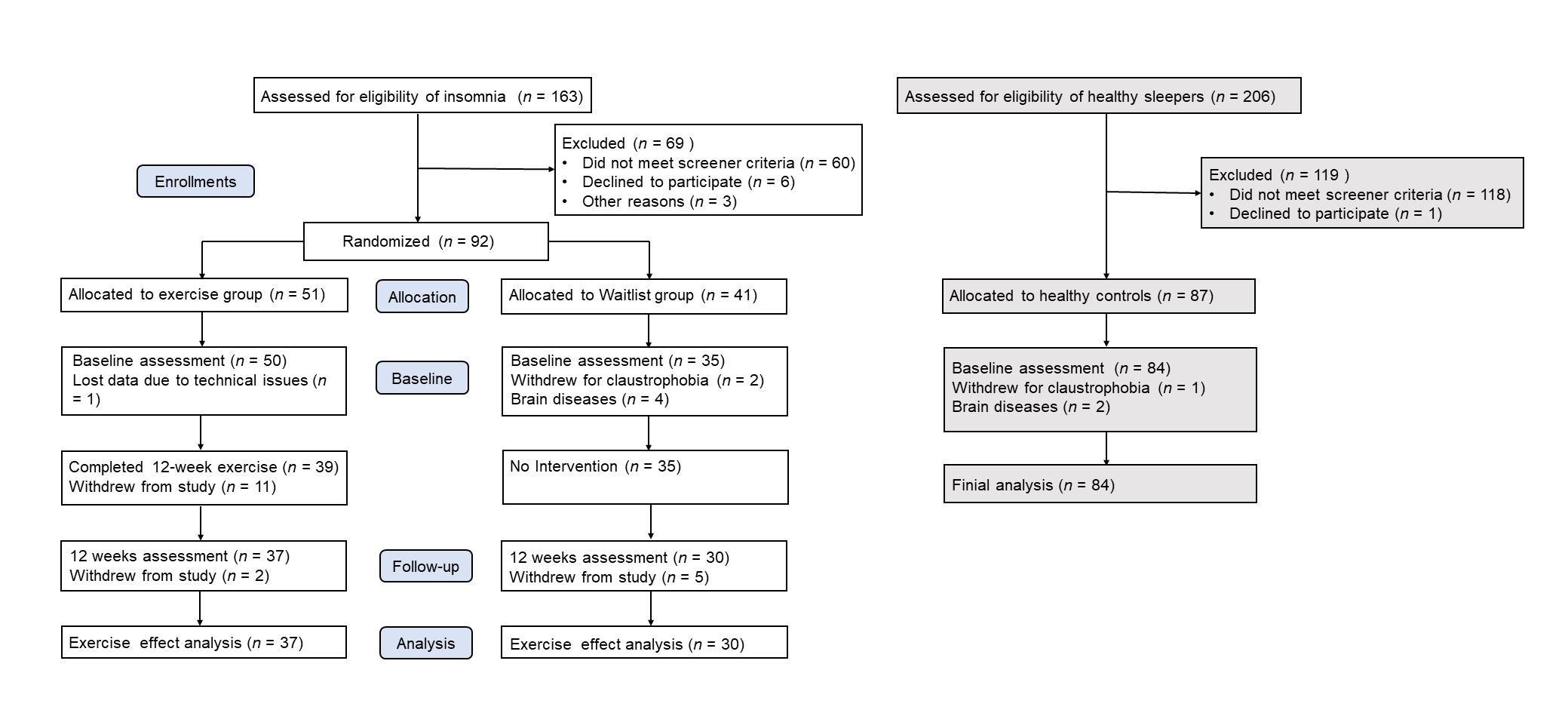

Supplement: Supplementary file 1 — Supplementary materials [file 41398_2024_2875_MOESM1_ESM.docx]
